# Mitigation potential of individual and combined dietary supplementation of local Bentonite Clay and Distillery Sludge against Ochratoxin-A induced toxicity in broilers

**DOI:** 10.1186/s12917-022-03466-3

**Published:** 2022-10-19

**Authors:** Mian Muhammad Awais, Ujala Mehtab, Muhammad Irfan Anwar, Muhammad Raza Hameed, Masood Akhtar, Ahmad Raza, Riffat Aisha, Faqir Muhammad, Muhammad Kashif Saleemi, Ahad Fayyaz

**Affiliations:** 1 Department of Pathobiology, Faculty of Veterinary Sciences, Bahauddin Zakariya University, Multan, Pakistan; 2 Department of Biosciences, Faculty of Veterinary Sciences, Bahauddin Zakariya University, Multan, Pakistan; 3 Department of Pathology, University of Agriculture, Faisalabad, Pakistan

**Keywords:** Ochratoxicosis, *Aspergillus ochraceus*, Immunity, Toxin adsorbents, Poultry

## Abstract

**Background:**

This study aimed to evaluate the ameliorative effects of dietary supplementation of local bentonite clay (BN) and distillery sludge (DS) alone and in combination on ochratoxin-A (OTA) induced toxicity in broilers. For this purpose, day-old-broiler chicks (*n* = 270) were procured from the local market and reared under standard management conditions. After 7 days of acclimatization, birds were divided into 2 main groups A and B with respect to OTA inclusion level in feed, each with four sub-groups viz. A1-A4, each challenged with OTA at a dietary inclusion level of 250 µg/kg feed and B1-B4, each challenged with OTA at the level of 500 µg/kg feed and a common control group that was fed with basal feed throughout the experiment. In groups A and B, BN and DS were administered with feed at the rate of 10 g/kg of feed and 5 g/kg of feed alone and in combination, respectively.

**Results:**

Results showed that OTA administration alone resulted in poor feed conversion ratio (FCR) and immunological responses along with increased serum levels of alanine transaminase (ALT), Aspartate transaminase (AST), urea and creatinine (*P* < 0.05). A significant decrease (*P* < 0.05) in serum protein levels (albumin, globulin and total protein) was also observed in OTA-fed groups in a dose-dependent manner. The addition of BN at 10 g/kg of OTA-contaminated feed resulted in better FCR and immunological responses as compared to those fed OTA only. The BN supplementation also conferred protection against elevation of serum biochemical parameters when compared with OTA-fed groups. However, the addition of DS could not provide significant protection (*P* > 0.05) on alteration of serum biochemical parameters in response to the OTA induced toxicity. The combined supplementation of BN and DS resulted in amelioration of OTA-induced toxicity and showed improved FCR, immunological, hematological and serum biochemical parameters (*P* < 0.05) when compared with other groups. Similarly, BN and DS resulted in a significant decline (*P* < 0.05) in the OTA tissue residues compared with other groups and control.

**Conclusion:**

In conclusion, combined dietary supplementation of BN (10 mg/kg) and DS (05 mg/kg) in feed reduced the toxic effects of OTA contamination at levels of 250 and 500 µg/kg of feed in broilers. So, the combination products of BN and DS may be successfully developed for use in poultry for protection against OTA-induced toxicity in broilers.

## Background

Fungi are multicellular organisms that are present globally on almost all artificially preserved agricultural products [1, 2]. Some species of fungi are notorious for producing toxins/toxic metabolites termed as mycotoxins [3]. Approximately more than 400 different types of mycotoxins have been identified worldwide [4]. Mycotoxins served as secondary metabolites in animal feedstuff, produced by filamentous molds, having a major role in contaminating the improperly stored feed [5]. Molds grow well in warm and humid conditions, contaminating the animal feed ingredients worldwide, especially cereals. Currently, ochratoxins (OT) are considered a serious threat for the poultry industry. These have been demonstrated not only for toxic effects on the well-being and performance of birds but also for having deleterious effects in humans in the form of Balkan endemic neuropathy and fatal kidney disease [6]. These are further classified into three types viz. OTA, OTB and OTC. Out of these, OTA is highly toxic as compared to other types [7]. The OTA is mainly produced by *Aspergillus* (*A*.) *ochraceus* and *Penicillium viridicatum*, although other species of *Aspergillus* like *A. alliaceous, A. malleus, A. obtains, A. partake, A. sclentiorum and A. sulphureus* had also been reported for producing OTA [8]. The OTA resulted in severe economic losses in the poultry industry due to its deleterious effects on the growth rate, feeding efficiency and high mortality [9]. Once a mycotoxin enters the food chain, its 100% eradication is not possible [10]. Previous literature regarding their toxicity testing revealed that LD_50_ (Oral median lethal dose) values in turkeys, quails and ducks and chicken were 5.9, 16.5, 0.5 and 2 to 4 mg/kg of body weight, respectively [11]. Absorption of OTA occurs in the gastrointestinal tract and binding with albumin enhances the half-life of OTA from a few days to months, depending upon specie [12]. The OTA produces toxicological effects like nephrotoxicity, hepatotoxicity and teratotoxicity both in animals and humans [13]. Previous studies revealed that OTA-induced vascular degeneration, hyperemia and pre-capillary edema of kupffer cells along with karyolysis in hepatocytes of affected broilers [14]. Apart from degenerative effects of OTA in kidneys and livers of broilers, it had also been reported for a negative impact on the development of lymphoid organs resulting in reduced organ-body weight ratios [15]. To avoid the undesired impact of mycotoxins, a variety of absorbents including different clays, distillery sludge (DS), activated charcoal and Vitamins including Vit. E and beta-glucans had been evaluated for ameliorative effects against mycotoxicosis in poultry birds [16].

The DS, a by-product of the sugar cane industry, is produced in bulk amounts with easy availability at cheaper rates. It contains proteins, different vitamins (ascorbic acid, etc.) and various essential amino acids including arginine, lysine and methionine, etc. The high protein contents along with essential vitamins in DS not only help in improving the growth performance of the birds and may also help in the detoxification of the adverse effects of mycotoxins by preventing denaturation of proteins and oxidative stress [7]. The BN clay is a phyllosilicate substance (a subtype of aluminosilicates) and contains a high level of montmorillonite. The BN reservoirs are available worldwide and are easily available at cheaper rates [17]. The binding potential of BN clay to various mycotoxins was previously studied and proved as a potent agent for adsorption of different mycotoxins including OTA [10]. The addition of such feed additives in contaminated feed results in selective binding of different mycotoxins and thus prevents their absorption from the digestive systems. Additionally, several other benefits including easy availability, low cost and eco-friendly make them ideal candidates for use in the poultry industry against mycotoxicosis.

Although studies are available on the individual use of BN and DS against mycotoxicosis but limited/no information is available on their combined effect against OTA-induced toxicity. Previous studies revealed that BN and DS when used alone provided partial protection against mycotoxicosis in poultry [2, 7, 10]. Keeping in view the different mechanisms of action of BN and DS, it was hypothesized that combined use of BN and DS might confer better protection as compared to their individual use through a synergistic effect.

Therefore, this study was conducted to assess the mitigation potential of individual and combined dietary supplementation of local bentonite (BN) and DS against toxicity induced by different dietary levels of OTA in broilers.

## Results

### Evaluation of growth performance

**Fig. 1:**
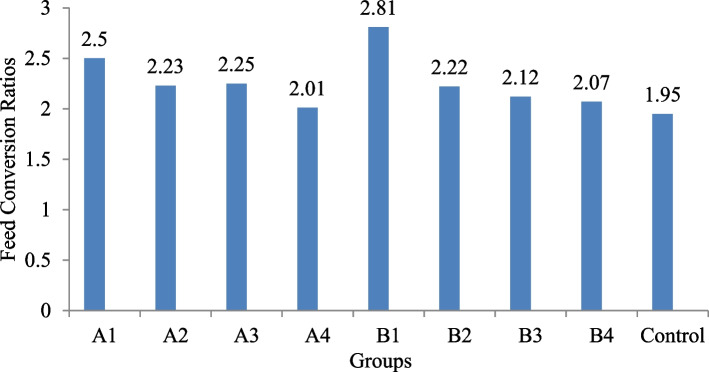
Feed conversion ratios in OTA-fed experimental and control groups. A1 (OTA 250 µg/kg), A2 (OTA 250 µg/kg + BN 10 g/kg), A3 (OTA 250 µg/kg + DS 5 g/kg), A4 (OTA 250 µg/kg + BN 10 g/kg + DS 5 g/kg), B1 (OTA 500 µg/kg), B2 (OTA 500 µg/kg + BN 10 g/kg), B3 (OTA 500 µg/kg + DS 5 g/kg), B4 (OTA 500 µg/kg + BN 10 g/kg + DS 5 g/kg), C (Control group)

The groups fed with OTA along with BN and DS showed comparatively better FCR when compared with groups fed with OTA alone. Further, it was also observed that groups fed with DS and BN in combination to counteract the OTA toxicity showed relatively improved FCR values (A4 = 2.01; B4 = 2.07) when compared with other treatment groups (A1-A3 and B1-B3) except control (1.95) group (Fig. 1).

### Serum biochemical parameters

Results indicated significantly (*P* < 0.05) elevated ALT value in group B1 fed OTA at an inclusion level of 500 µg/kg of feed only as compared to all other treatment and control groups except B2, B3 and A1. On the other hand, group A1 also showed a non-significant difference (*P* > 0.05) with all other treatment groups except the control. The AST value was significantly elevated in group B1 (OTA; 500 µg/kg of feed) as compared to all other treatments and control groups. The groups fed OTA at a dietary level of 250 µg/kg of feed showed significantly lower (*P* < 0.05) AST values compared to those fed OTA at a dietary level of 500 µg/kg of feed. In groups fed dietary OTA at the inclusion level of 500 µg/kg of feed, the addition of DS alone or in combination with BN significantly (*P* < 0.05) improved AST values as compared to those administered with BN alone. The urea level was significantly elevated (*P* < 0.05) in group B1 as compared to all other groups. At dietary level of 250 µg OTA/kg of feed, the groups administered with BN and DS alone or in combination showed non-significant difference (*P* > 0.05) of urea levels compared to the control. In groups fed OTA at the level of 500 µg /kg of feed, those administered with DS alone or in combination with BN showed significantly lower urea level compared to groups administered with BN alone. In contrast, a non-significant difference was observed in creatinine levels in treatment and control groups (*P* > 0.05). The total protein level was significantly decreased (*P* < 0.05) in group B1, whereas non-significant differences were observed in all other treatment groups. Almost similar trend was observed for serum albumin and globulin levels in treatment and control groups (Table 1).

**Table 1:** Serum biochemical profiles (Mean ± SEM) of OTA-induced experimental and control groups

**Group**	**ALT(U/L)** **(Mean ± SEM)**	**AST(U/L)** **(Mean ± SEM)**	**Urea** **(mg/100 ml)** **(Mean ± SEM)**	**Creatinine** **(mg/100 ml)** **(Mean ± SEM)**	**Total Proteins** **(g/dl)** **(Mean ± SEM)**	**Globulins** **(g/dl)** **(Mean ± SEM)**	**Albumin** **(g/dl)** **(Mean ± SEM)**
**A**_**1**_	35.0 ± 12.1^ab^	164 ± 12.1^c^	31.0 ± 3.00^c^	0.31 ± 0.09^ab^	3.10 ± 0.36^bc^	1.10 ± 0.26^b^	2.50 ± 0.10^ef^
**A**_**2**_	26.0 ± 6.24^bc^	144 ± 7.51^de^	23.0 ± 3.00^de^	0.27 ± 0.04^ab^	3.57 ± 0.51^ab^	0.93 ± 0.12^b^	2.83 ± 0.21^de^
**A**_**3**_	21. 7 ± 3.06^bc^	132 ± 4.73^e^	18.7 ± 2.08^e^	0.28 ± 0.10^ab^	3.23 ± 0.21^b^	0.70 ± 0.30^bc^	3.20 ± 0.17^bc^
**A**_**4**_	26.3 ± 13.8^bc^	127 ± 16.5^e^	18.0 ± 2.00^e^	0.23 ± 0.29^a^	2.90 ± 0.70^bc^	0.83 ± 0.21^a^	0.30 ± 0.30^bc^
**B**_**1**_	46.0 ± 13.1^a^	212 ± 11.5^a^	56.3 ± 8.39^a^	0.45 ± 0.05^a^	2.47 ± 0.45^c^	0.33 ± 0.23^c^	2.33 ± 0.23^f^
**B**_**2**_	36.3 ± 4.16^ab^	187 ± 6.51^b^	47.7 ± 7.23^b^	0.32 ± 0.07^ab^	3.07 ± 0.12^bc^	0.83 ± 0.21^b^	3.03 ± 0.15^bcd^
**B**_**3**_	32.3 ± 2.08^abc^	161 ± 9.61^c^	31.7 ± 3.06^c^	0.31 ± 0.08^ab^	3.10 ± 0.25^bc^	0.77 ± 0.25^bc^	2.97 ± 0.06^ cd^
**B**_**4**_	24.0 ± 9.54^bc^	153 ± 11.6^ cd^	28.3 ± 5.13^ cd^	0.30 ± 0.10^ab^	3.33 ± 0.45^ab^	0.93 ± 0.06^b^	3.33 ± 0.25^b^
**Control**	19.3 ± 4.04^c^	129 ± 4.04^e^	16.3 ± 1.53^e^	0.23 ± 0.06^a^	4.03 ± 0.45^a^	1.60 ± 0.53^a^	3.80 ± 0.20^a^

A1 (OTA 250 µg/kg), A2 (OTA 250 µg/kg + BN 10 g/kg), A3 (OTA 250 µg/kg + DS 5 g/kg), A4 (OTA 250 µg/kg + BN 10 g/kg + DS 5 g/kg), B1 (OTA 500 µg/kg), B2 (OTA 500 µg/kg + BN 10 g/kg), B3 (OTA 500 µg/kg + DS 5 g/kg), B4 (OTA 500 µg/kg + BN 10 g/kg + DS 5 g/kg), C (Control group); For each parameter, values sharing similar superscripts do not differ significantly (*p* > 0.05)

### Evaluation of immune responses

#### Lymphoblastogenic responses to PHA-P

Results showed that lymphoblastogenic response to PHA-P was significantly decreased (*P* < 0.05) in all treatment groups compared with control at 24, 48 and 72 h post-PHA-P injection. The sub-group B1 administered with OTA at the level of 500 µg/kg of feed showed significantly lower (P < 0.05) lymphoblastogenic response than all other experimental and control groups. The results indicated that at 24 and 48 h, groups administered with BN and DS in combination (A4 and B4) showed statistically similar responses (*P* > 0.05) as compared to control, but their difference was significantly higher (*P* < 0.05) as compared to birds of groups administered with DS or BN alone. After 72 h post-PHA-P injection, all the experimental groups except B1 showed non-significant differences compared to the control (Table 2).

**Table 2:** Lymphoblastogenic response to PHA-P in OTA-induced experimental and control groups

**Group**	**Time Post-PHA-P Injection**		
	**24 Hours** **(Mean ± SEM)**	**48 Hours** **(Mean ± SEM)**	**72 Hours** **(Mean ± SEM)**
**A**_**1**_	0.18 ± 0.03^de^	0.17 ± 0.05^bcd^	0.13 ± 0.06^bc^
**A**_**2**_	0.24 ± 0.04^bcd^	0.21 ± 0.02^abc^	0.14 ± 0.07^bc^
**A**_**3**_	0.23 ± 0.04^ cd^	0.17 ± 0.03^bc^	0.15 ± 0.05^bc^
**A**_**4**_	0.30 ± 0.03^ab^	0.21 ± 0.05^abc^	0.17 ± 0.04^abc^
**B**_**1**_	0.13 ± 0.04^e^	0.11 ± 0.05^d^	0.11 ± 0.03^c^
**B**_**2**_	0.23 ± 0.06^ cd^	0.15 ± 0.03^ cd^	0.15 ± 0.03^bc^
**B**_**3**_	0.22 ± 0.02^d^	0.19 ± 0.02^bc^	0.16 ± 0.04^abc^
**B**_**4**_	0.29 ± 0.03^abc^	0.22 ± 0.03^ab^	0.20 ± 0.01^ab^
**Control**	0.35 ± 0.05^a^	0.27 ± 0.03^a^	0.21 ± 0.03^a^

A1 (OTA 250 µg/kg), A2 (OTA 250 µg/kg + BN 10 g/kg), A3 (OTA 250 µg/kg + DS 5 g/kg), A4 (OTA 250 µg/kg + BN 10 g/kg + DS 5 g/kg), B1 (OTA 500 µg/kg), B2 (OTA 500 µg/kg + BN 10 g/kg), B3 (OTA 500 µg/kg + DS 5 g/kg), B4 (OTA 500 µg/kg + BN 10 g/kg + DS 5 g/kg), C (Control group); Mean values sharing similar superscripts in a coumn do not differ significantly (*p* > 0.05)

#### Macrophage activity by carbon clearance assay

**Fig. 2:**
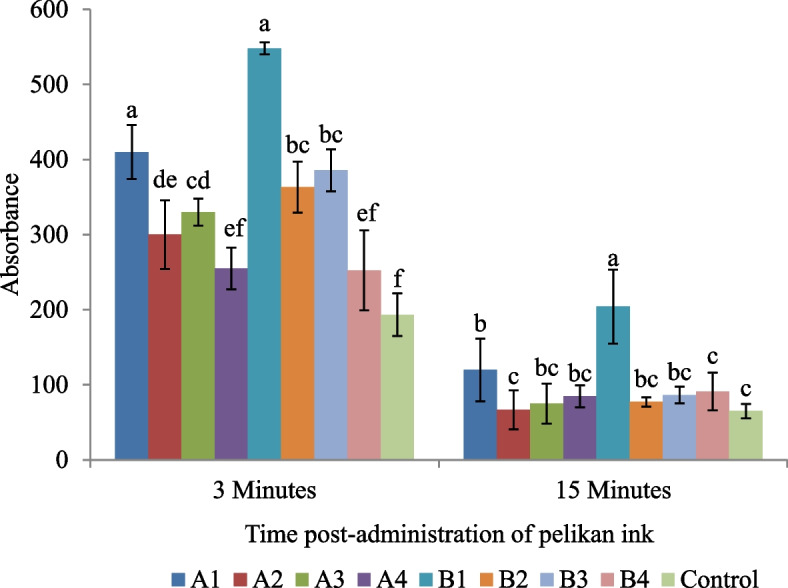
Phagocytic response by carbon clearance assay in OTA-fed experimental and control groups. A1 (OTA 250 µg/kg), A2 (OTA 250 µg/kg + BN 10 g/kg), A3 (OTA 250 µg/kg + DS 5 g/kg), A4 (OTA 250 µg/kg + BN 10 g/kg + DS 5 g/kg), B1 (OTA 500 µg/kg), B2 (OTA 500 µg/kg + BN 10 g/kg), B3 (OTA 500 µg/kg + DS 5 g/kg), B4 (OTA 500 µg/kg + BN 10 g/kg + DS 5 g/kg), C (Control group); Bars sharing similar letters at specific time intervals are statically non-significant (*P* > 0.05)

Results showed the highest macrophage activity in the control group at 3 min post-Pelikan injection, whereas the difference of control group with A4 and B4 groups administered with BN and DS in combination was statistically non-significant (*P* > 0.05). On the other hand, at 15 min, the OD values showed non-significant differences between all the treatment and control groups except B1 (OTA; 500 µg/kg of feed only) which showed significantly (*P* < 0.05) lowest carbon clearance activity as depicted by highest OD value (Fig. 2).

#### Antibody responses to Sheep erythrocytes (SE)

At both days 7^th^ and 14^th^ post-SE injection, the highest geometric mean titres for total Immunoglobulins (Ig) were recorded in the healthy control group (C) followed by those administered with BN and DS in combination at both dietary levels i.e. 250 and 500 µg/kg of feed. A similar pattern was recorded for IgM and IgG titers on 7^th^ and 14^th^ post-SE injection. It was also observed that groups administered with BN and DS in combination showed better humoral immune responses (total Ig, IgM and IgG) at both dietary levels i.e., 250 and 500 µg/kg of feed compared to those administered with BN or DS, alone (Table 3).

**Table 3:** Antibody response to sheep erythrocytes (SE) in OTA-induced experimental and control groups

**Group**	**Total Igs**	**IgM**	**IgG**
**Day 7 Post-SE Injection**			
**A**_**1**_	80.6	48.6	32.0
**A**_**2**_	102	69.6	32.0
**A**_**3**_	102	61.3	40.3
**A**_**4**_	161	111	50.8
**B**_**1**_	50.8	38.8	16.0
**B**_**2**_	64.0	43.8	20.2
**B**_**3**_	80.6	55.2	25.4
**B**_**4**_	128	87.7	40.3
**Control**	323	242	80.6
**Day 14 Post-SE Injection**			
**A**_**1**_	50.8	18.8	32.0
**A**_**2**_	64.0	23.7	40.3
**A**_**3**_	80.6	29.8	50.8
**A**_**4**_	102	37.6	64.0
**B**_**1**_	40.3	18.9	25.4
**B**_**2**_	50.8	25.4	25.4
**B**_**3**_	64.0	23.7	40.3
**B**_**4**_	102	37.6	64.0
**Control**	256	98.7	161

A1 (OTA 250 µg/kg), A2 (OTA 250 µg/kg + BN 10 g/kg), A3 (OTA 250 µg/kg + DS 5 g/kg), A4 (OTA 250 µg/kg + BN 10 g/kg + DS 5 g/kg), B1 (OTA 500 µg/kg), B2 (OTA 500 µg/kg + BN 10 g/kg), B3 (OTA 500 µg/kg + DS 5 g/kg), B4 (OTA 500 µg/kg + BN 10 g/kg + DS 5 g/kg), C (Control group)

#### Detection of OTA residues in liver tissues

**Fig. 3:**
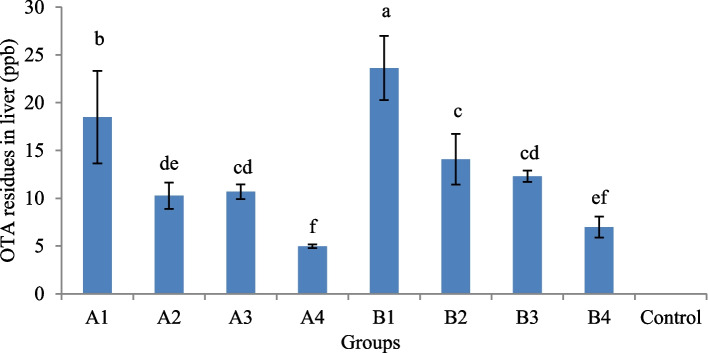
Levels of OTA residues in liver tissues of OTA-fed experimental and control groups. A1 (OTA 250 µg/kg), A2 (OTA 250 µg/kg + BN 10 g/kg), A3 (OTA 250 µg/kg + DS 5 g/kg), A4 (OTA 250 µg/kg + BN 10 g/kg + DS 5 g/kg), B1 (OTA 500 µg/kg), B2 (OTA 500 µg/kg + BN 10 g/kg), B3 (OTA 500 µg/kg + DS 5 g/kg), B4 (OTA 500 µg/kg + BN 10 g/kg + DS 5 g/kg), C (Control group); Bars with different superscripts differ significantly (*P* < 0.05)

The results showed that no detectable OTA residues were found in liver tissue of control group. However, OTA residues were detected in all other treatment groups with significantly highest values (*P* < 0.05) in liver tissues of the group B1 administered with an OTA inclusion level of 500 µg/kg of feed, followed by group A1 administered with OTA inclusion level of 250 µg/kg of feed. Among OTA-fed groups, A4 and B4 administered with BN and DS in combination showed significantly lower (*P* < 0.05) residue levels as compared to groups administered with anyone of BN or DS; however, the differences in groups administered with BN and DS alone were statistically non-significant (*P* > 0.05) (Fig. 3).

#### Histopathological findings

##### Kidneys

**Fig. 4:**
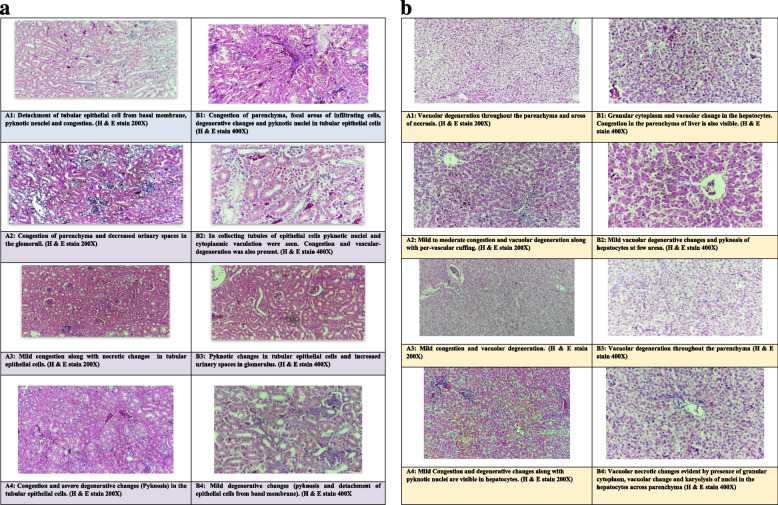
**a**. Photomicrographs of Kidney tissues of broilers of different groups fed OTA alone and in combination with Bentonite clay and Distillery sludge.A1 (OTA 250µg/kg), A2 (OTA 250µg/kg + BN 10g/kg), A3 (OTA 250µg/kg + DS 5g/kg), A4 (OTA 250µg/kg + BN 10g/kg + DS 5g/kg), B1 (OTA 500 µg/kg), B2 (OTA 500µg/kg + BN 10g/kg), B3 (OTA 500µg/kg + DS 5g/kg), B4 (OTA 500µg/kg + BN 10g/kg + DS 5g/kg), C (Control group). **b**. Photomicrographs of liver tissues of broilers of different groups fed OTA alone and in combination with Bentonite clay and Distillery Sludge. A1 (OTA 250µg/kg), A2 (OTA 250µg/kg + BN 10g/kg), A3 (OTA 250µg/kg + DS 5g/kg), A4 (OTA 250µg/kg + BN 10g/kg + DS 5g/kg), B1 (OTA 500 µg/kg), B2 (OTA 500µg/kg + BN 10g/kg), B3 (OTA 500µg/kg + DS 5g/kg), B4 (OTA 500µg/kg + BN 10g/kg + DS 5g/kg), C (Control group)

Congestion and tubular epithelial necrotic changes were observed in the tissue parenchyma. Detachment of tubular epithelial cells from the basal membrane and degeneration (pyknotic nuclei) were also commonly observed in the birds fed with OTA at an inclusion level of 250 µg/kg of feed. Shrinkage of glomeruli in different areas of parenchyma (kidney) was also observed in the birds belonging to this group. Moderate to severe necrotic and degenerative changes were observed in the kidneys of the birds fed with OTA 500 µg/kg of feed. Widespread congestion was detected in most of the sections with necrotic epithelial cells of proximal convoluted tubules in the kidneys. Detachment of tubular epithelial cells was a severe and more common feature in groups fed with OTA only (A1 and B1) compared to all other groups. Infiltrations of inflammatory cells were also observed in a few sections of this group. However, these changes were mild to moderate in groups fed with BN and DS sludge alone or in combination when compared with control and only OTA-fed groups (A1 and B1) (Fig. 4a).

##### Liver

Widespread degenerative changes in the liver parenchyma were observed in most of the tissue sections. These changes were evident from vacuolar degeneration and nuclear changes (pyknosis, karyorrhexis and karyolysis). Infiltrating cells around blood vessels were commonly observed along with mild to moderate congestion in treatment groups that were fed OTA-contaminated feed at dietary levels of 250 µg OTA/kg of feed. These changes were severe and more frequent in groups fed with 500 µg OTA/kg of feed. It was also observed that the addition of BN and DS (alone or in combination) in OTA-contaminated feed resulted in a marked decrease in the severity and consistency of these lesions compared with birds administered OTA-contaminated feed only (Fig. 4b).

## Discussion

The worldwide presence of OTA and its excellent thermostability is a big challenge for its eradication from the food web [18]. In this study, two different levels of OTA-contaminated poultry feeds (250 and 500 µg/kg) were used to elucidate the mitigation potential of BN and DS against ochratoxicosis in poultry.

The BN is a phyllosilicate substance, a subtype of aluminosilicates with the major montmorillonite component. The BN reservoirs are available worldwide, but BN contents can vary and may alter the absorption of nutrients at higher levels [19, 20]. The binding potential of BN clay to various mycotoxins was previously studied and proved as a potent agent for adsorption of different mycotoxins, including OTA [21]. The DS is a waste product of the sugar industry obtained after the fermentation of molasses. *Saccharomyces cerevisiae* is a major component of DS which contains 85–90% polysaccharide fraction (β-D-glucans). These polysaccharide fractions are actively involved in the binding of mycotoxins [10, 22]. The DS contains heavy metal contents which affect the activity of microbial enzymes [23]. The easy availability and binding capacity of BN and DS make them potential candidates for the amelioration of toxic and pathological effects of OTA in broilers.

The present study described that feeding OTA-contaminated feed to broilers had negatively influenced FCR and thus feeding efficiency in a dose-dependent manner in accordance with the findings of previous similar studies [24, 25]. Addition of BN at 10 g/kg of feed resulted in better FCR when compared with the group fed OTA alone. Conversely, Santin and his co-workers [26] reported that hydrated sodium calcium aluminosilicates (HSCAS; an adsorbent supplemented at a dietary level of 0.25% w/w) could not prevent the toxic effects of OTA at the level of 2 mg/kg of feed in broilers. However, the addition of 0.2% HSCAS along with OTA at the level of 68.4 µg/kg of feed resulted in a non-significant difference in feed intake and average daily weight gains, compared with control group in broilers [27] which might be due to low levels of OTA in the diet.

The results of FCR did not conclude better response by using DS to prevent OTA-induced adverse effects in the present study compared with control. However, improved FCR was observed compared with groups fed OTA (alone). These results are in line with previous findings that reported increased feed intake by adding DS in broiler diet. The higher feed intake in birds fed diets containing DS (1.5 and 3.0% w/w) might be due to the presence of vitamins, especially B-complex, oligomonosaccharides and other bioactive nutrients, which increased the palatability of the diet [28]. Similarly, esterified glucomannan and BN in diet had also been reported to ameliorate the performance framework of birds affected by OTA [10, 29]. However, separate use of BN and DS for mitigating OTA toxic effects did not result in better FCR during the present study. Contrary to these, the combination of BN and DS resulted in amelioration of OTA toxicity and showed improved FCR close to the control group fed basal diet only. It might be due to the additive effect of DS on the adsorptive capabilities of BN with better intestinal absorption when used in combination.

The OTA feeding in broiler birds resulted in increased serum levels of ALT, AST, urea and creatinine in the present study. These effects were persistent and observed in dose dependent-manner in groups fed with OTA at dietary levels of 250 and 500 µg/kg of feed. These findings were in linewith the earlier reports describing OTA toxic effects in poultry [14, 15, 29–31]. The increased AST level in the present study could be attributed to enzyme leakage in response to OTA-induced degenerative changes in hepatocytes, as shown previously [31] and indicated liver damage. The increased serum levels of ALT and AST indicate hepatic damage and degeneration in the liver tissue. They are considered indicators for cell viability improvements and cell membrane permeation due to hepatic disruption. The serum protein levels (albumin, globulin and total proteins) of broilers exposed to OTA-contaminated feed were significantly decreased in the current study. Similarly, some previous studies had also reported a decrease in serum protein levels in broiler chicks fed with OTA-contaminated diet [26, 31]. The decreased serum protein levels might be attributed to inhibition of protein synthesis due to OTA toxicity as one of its major adverse outcomes [24].

In the present study, elevated serum biochemical parameters were observed in the group fed with OTA alone but the difference was non-significant when compared with groups fed with BN-supplemented diet in addition to OTA. Similar findings on ameliorative effects of different clays against OTA-induced toxicity had been reported previously [10, 21]. The present study indicated that adding BN could partially prevent the alteration of serum biochemical parameters but did not offer complete protection against OTA-induced toxicity.

Supplementation of DS in the diet of birds did not result in any significant protection on alteration of serum biochemical parameters in response to the OTA-induced toxicity during this study. These results were in accordance with the findings of Khatoon et al. [7, 10], who reported partial protection against immunological and serum biochemical parameters in broilers by using DS to ameliorate the OTA-induced toxicity at a dose rate of 0.3 mg/kg of feed. However, the combined use of DS and BN resulted in a synergistic effect and significantly protected the physiological levels of serum enzymes in the current study.

A decrease in humoral and cell-mediated immune responses was observed in groups fed with OTA (alone) compared to other treatment groups fed with BN and DS along with OTA. Some previous studies [24, 25] also reported similar OTA-induced immunosuppression in broilers fed with diets containing OTA in a dose-dependent manner. Some earlier studies had also reported OTA dependent decrease in immune responses in broiler and layer birds that might be attributed to adverse effects of OTA on immune organs resulting in decreased size of organs and number of immune cells [32–34]. Lower antibody titer might also be correlated with reduced protein synthesis in OTA-fed broilers.

Findings of the present study showed that the inclusion of BN clay in feed resulted in better immunological responses in terms of antibody titers to SEs, lymphoblastogenic activity to PHA-P and *in-vivo* phagocytic activity towards CCA compared with OTA-fed groups. However, complete mitigation of OTA-induced immunosuppressive effects could not be observed in the present study. Previous studies also demonstrated comparable effects using avian tuberculin (as mitogen) in chicken and described mild or no ameliorative effect of BN on immune-related parameters due to OTA-induced toxicity. Our results are consistent with the findings of Santin et al. [26], who reported that dietary inclusion of OTA (2 ppm) reduced both humoral and cellular immune responses in broilers and inhibited phagocytosis that could be mitigated by the addition of BN at the dietary inclusion level of 0.25%.

In the present study, the inclusion of DS in feed resulted in increased cellular and humoral immune response in broilers with experimentally induced ochratoxicosis. A similar study [35] described the partial ameliorative activity of DS against OTA-induced toxicity, which resulted in the prevention of immunosuppressive effects. Overall, combined use of BN and DS resulted in better immune responses in broilers with experimentally induced OTA toxicity at different dietary levels.

Gross and histopathological lesions in different organs of broiler birds fed with OTA-contaminated feed were observed in line with earlier findings [14, 24, 36–38]. Persistent histopathological findings in the liver of broiler groups fed with OTA included cellular swelling, vacuolation, pyknosis, congestion and infiltration of cells around blood vessels in liver parenchyma as a prominent feature of necrotic and degenerative changes. While in kidneys, cellular swelling, necrosis of tubular epithelial cell and infiltration of inflammatory cells in different areas of kidney parenchyma were persistent histopathological findings in OTA-fed groups. However, in addition to these, proliferation of bile duct had also been reported in liver of birds fed with OTA-contaminated feed [39]. Elaroussi et al. [32] described no vacuolar degeneration in hepatocytes contrary to the degenerative vacuolar changes in the liver tissues of broilers fed with OTA (0.25 and 0.5 mg/kg) as observed in this study. Contrary to our findings, fibrosis in focal areas and around the bile duct had also been reported following dietary exposure of 0.354 mg/kg OTA in broiler chickens [39]. The use of BN and DS decreased the severity and consistency of histopathological changes associated with OTA-induced toxicity when used alone however, their protective effect was more pronounced when used in combination. These findings indicated that BN and DS may give partial protection against OTA-induced hepatic and nephrotoxicity but failed to offer complete protection at dose levels used in the current study. Khan et al. [35] also reported similar findings using BN at lower levels, but limited information is available regarding protection by using DS in poultry to prevent OTA-induced degenerative changes. The current study is a valuable addition to existing literature highlighting the protective effects of DS alone or in combination with BN against histopathological changes induced by OTA at different dietary levels in broilers.

The lower levels of OTA residues were observed in the liver tissues of birds supplemented with BN and DS along with different dietary levels of OTA when compared with those fed with OTA-contaminated diets only. The presence of tissue residues of OTA in the liver of birds exposed to OTA-contaminated diet is in line with findings of previous studies [15, 24, 36, 39]. The higher OTA residue levels in liver tissues of birds fed with OTA-contaminated diets might be due to the metabolism and excretion of OTA through the hepatobiliary system [39]. It had also been reported that dietary supplementation of HSCAS (1%) and acid bentonite (1%, 10%) to OTA-contaminated feed (1.0 mg/kg) did not affect the blood or tissue levels of the toxins in pigs [40]. However, the present study showed that the use of BN and DS in combination resulted in a significant decrease in the tissue residues of OTA compared with other groups. It might be hypothesized that combined use of BN and DS resulted in decreased availability of OTA in target organs, resulting in better ameliorative effects against OTA-induced toxicity compared to their individual use.

In short, dietary addition of BN alone and/or its related compounds could only partially protect against OTA-induced toxic effects in broilers. On the other hand, limited information is available on the use of DS concerning its optimal dosage for ameliorative effects against different dietary levels of OTA, although such studies also reported partial protection against mycotoxicosis [35]. Although we used different levels of BN and DS as mentioned in previous studies where they were used separately, but there is need to optimize their inclusion levels in feed when used in combination to get optimal protection against ochratoxicosis.

In conclusion, the combined use of DS and BN showed mitigation potential against OTA-induced toxicity in broilers and thus has the potential to be commercialized. Further studies on the characterization, dose standardization, field trials and commercial feasibility are underway to avoid economic losses associated with ochratoxicosis in the poultry industry.

## Methods

### Production and quantification of OTA

Pure culture of *A. ochraceus* was obtained from Toxicopathological Lab, Department of Pathology, University of Agriculture Faisalabad-Pakistan from which OTA was produced on wheat grains in One Health Research Lab, Department of Pathobiology, Bahauddin Zakariya University (BZU), Multan-Pakistan according to the methodology described previously [41] with minor modifications [24, 25]. The OTA was extracted from fermented wheat grains and quantified using High Performance Liquid Chromatography (HPLC) according to the previously described methodology [8]. Basal diet for the experiment was prepared without growth promotors and other feed additives, including toxin binders. Basal diet was used to prepare OTA-contaminated feeds at required concentrations i.e. 250 and 500 µg/kg, using the methodology described earlier [25], and were subjected to HPLC analysis to ensure the required dietary concentration in the feed.

### Procurement of DS and BN clay

The DS was obtained from Tandliawala Sugar Mill, Muzaffar-Garh, Punjab-Pakistan and BN clay was obtained from the local market of Multan, Punjab-Pakistan.

### Experimental design

One-day-old broiler chicks (Cobb; *n* = 270) were purchased from the local market and raised under standard-management conditions at Experimental Poultry Shed, Faculty of Veterinary Sciences, BZU, Multan-Pakistan. The birds were reared on the floor system using wood shaving as litter. All the chicks were offered basal feed (Table 4) and vaccinated according to local vaccination schedule [42]. Prior approval was obtained from the Board of Studies of the Department of Pathobiology-BZU, followed by the Advanced Studies and Research Board of BZU, Multan.

**Table 4:** Composition of Basal diet used in the experiment

**Chemical Analysis**	**g/Kg**
Crude Protein	200.0
Crude Fat	45.0
Carbohydrates	420.0
Fiber contents	50.0
Lysine	12.0
Ash	55.0
Calcium	10.0
Phosphorus	5.0
Sodium	1.5
Methionine + Cystine	7 .0
Methionine	4.0

After acclimatization period of 7 days, chicks were divided into 3 major groups A (*n* = 120), B (*n* = 120) and C (Control; *n* = 30). The groups A and B were further subdivided into four equal sub-groups A1-A4 and B1-B4, each sub-group containing 30 chicks. The treatment groups were administered with OTA along with BN and DS as follows:

- A1 = OTA 250 µg/kg of feed
- A2 = OTA 250 µg/kg + BN 10 g/kg of feed
- A3 = OTA 250 µg/kg + DS 5 g/kg of feed
- A4 = OTA 250 µg/kg + BN 10 g/kg + DS 5 g/kg of feed
- B1 = OTA 500 µg/kg of feed
- B2 = OTA 500 µg/kg + BN 10 g/kg of feed
- B3 = OTA 500 µg/kg + DS 5 g/kg of feed
- B4 = OTA 500 µg/kg + BN 10 g/kg + DS 5 g/kg of feed
- C = Served as control group and fed on basal diet only

### Evaluation of growth performance

The growth performance was quantified in terms of FCR at the end of the experiment. Feeding efficiency of all groups was calculated by using the formula as follows: *FCR* = *feed consumed (gms)/weight gain (gms).*

### Evaluations of serum biochemical parameters

Blood samples were collected from wing veins of birds of all the groups and their sera were separated using serum harvesting tubes for serum biochemical analysis. Serum biochemical parameters including total proteins, albumin, ALT, AST, blood urea nitrogen (BUN) and Creatinine were determined by using commercially available kits (Innoline, Merck (Private) Limited-Pakistan; Product Codes: INO-17634, INO-17620, INO-17534, INO-17524, INO-17610 and INO-17554, respectively). While, serum globulin level was calculated by subtracting albumin concentration from total serum protein concentration.

### Evaluation of immune responses

#### Lymphoblastogenic response to Phyto-hemagglutinin-P (PHA-P)

The lymphoblastogenic response to PHA-P was quantified by classical toe web assay [43, 44]. Briefly, PHA-P (100 μg/100μL/bird) was injected in the interdigital space between 3^rd^ and 4^th^ digit of the right foot of birds on day 21^st^ of age, whereas the left foot was kept as control and injected with 0.1 ml of PSS (physiological saline solution). The lymphoblastogenic response was determined by subtracting the thickness of interdigital spaces of left foot from that of the the right foot at 24, 48 and 72 h post-PHA-P injection.

#### *Macrophage activity by* carbon clearance assay

Birds from each group were used to assess the competency of macrophages circulating in blood by using carbon clearance assay [45]. Briefly, Pelikan ink (1 mL/kg BW) was injected in birds of all experimental and control groups via the intra-venous route. Blood samples in EDTA-coated vacutainers were collected at pre (0 min) and post-Pelikan ink injection at 3 and 15 min. The blood plasma samples of birds from different groups were subjected to spectrophotometry to determine their optical density (OD) at a wavelength of 640 nm. The concentration of un-phagocytized carbon particles was assessed by calculating the difference of OD values at 0, 3 and 15 min by using the formula as follows:

$$\mathrm{Increased}\;\mathrm{absorbance}\left(\%\right)=\frac{\mathrm{Absorbance}\;\mathrm{post}\;-\;\mathrm{ink}\;\mathrm{injection}\;-\;\mathrm{Absorbance}\;\mathrm{pre}\;-\;\mathrm{ink}\;\mathrm{injection}}{\mathrm{Absorbance}\;\mathrm{pre}\;-\;\mathrm{ink}\;\mathrm{injection}}$$

#### Antibody response against sheep erythrocytes (SE)

Broilers from all the experimental and control groups were assessed for elicited humoral immune responses by using microplate hemagglutination assay. Briefly, a suspension of 3% washed SE (non-pathogenic T-cell mitogens) was injected intramuscularly (I/M) in broilers of all experimental and control groups at the rate of 1 mL per bird. Later, on days 7^th^ and 14^th^ post-SE-injection, sera samples were collected from broilers and titrated against SE to determine the total, IgG and IgM titers as described previously [42] and results were expressed as geometric mean titers (GMT).

#### Histopathological examination

Tissue samples from livers and kidneys from each group were collected at the last day of the experiment and preserved in a 10% neutral buffered formalin solution for histopathological examination using H&E (Hematoxyline & Eosin) staining method [46].

#### Evaluation of tissue residues

Tissue samples of livers were collected and preserved at -20 °C. Residues of OTA in the liver were assessed using a commercially available ELISA kit (Romer Labs®, Austria) by following the instructions of the manufacturer.

### Statistical analysis

The data obtained were analyzed by analysis of variance (ANOVA) using the MSTATC statistical software package. Duncan’s Multiple Range test was used to compare the means of different groups [47]. The differences were considered significant at *P* < 0.05.

## Data Availability

All data generated or analysed during this study are included in this published article. And further if required any other information related with the data involving in the manuscript can be obtained from the corresponding author upon reasonable request.
